# Mental health issues linked selfie addiction among South Indian college students

**DOI:** 10.6026/973206300210001

**Published:** 2025-01-31

**Authors:** Baskaran M., Riya Jacob, Jeyadeepa R., Raghuthaman G., Jayasudha A.

**Affiliations:** 1 Department of Mental Health Nursing, PSG College of Nursing, Coimbatore & The Tamil Nadu Dr. M.G.R. Medical University, Chennai, India; 2 Department of Psychiatric Nursing, Rajalakshmi College of Nursing, Chennai, India; 3 Department of Medical Surgical Nursing, PSG College of Nursing, Coimbatore, India; 4 Department of Psychiatry, Institute of Medical Sciences and Research, Coimbatore, India; 5 Department of Obstetrics and Gynecological Nursing, Apollo College of Nursing Hyderabad

**Keywords:** Mental health, pathway, selfie addiction, selfies, behaviour

## Abstract

Selfie addiction is a behaviour involving excessive selfie taking and sharing to obtain validation and acceptance on social media and it has been understudied. We used a simple random sampling method to recruit 165 students who were assessed for selfie addiction behaviour using Selfitis Behaviour Scale (SBS) before and after undertaking lecture series about selfie addiction. Data shows a significant reduction (t=12.119, p=0.0001) in mean SBS scores post-test (38.35±10.69) when compared to pre-test (57.20±18.44). Female gender was significantly associated with intensity of selfie addiction behaviour pre-test (p=0.0001), number of selfies clicked per day (p=0.0001) and where the selfies are posted (p=0.007). Thus, acute selfie addiction behaviour was manifested by most students which reduced significantly with teaching strategies.

## Background:

Smartphone addiction involves excessive use of social media sites for sharing selfies, leading to seeking validation, attention and self-objectification. It is considered a form of technological addiction [1]. A survey of 368 college students revealed positive correlations among selfies, body image satisfaction, self-objectification and narcissistic personality [2]. Selfie addiction behaviour analyzed 159 selfie victims from 111 incidents, focusing on selfie-related injuries and deaths. Results showed that most selfie victims were students, with incidents most common in India, US and Russia [3]. In India, studies conducted in Karnataka, Orissa and Mumbai reported selfie taking to be addictive among youngsters and adolescents and noted an 8.74% prevalence of dangerous selfies [4]. This study investigates the presence of selfie addiction behaviour among young adults. Apart from the physical dangers involved in social media selfie "challenges", where people even risk their lives to take these challenges, [5] selfie addiction allegedly impacts psychological wellbeing as reported in a study where young women were anxious, less confident, had concerns regarding their appearances, weight and shape, an overall dissatisfaction with their body and idealizing thin body image that even led to eating disorders [6]. A cross-sectional study was conducted on internet addiction among 244 students found a significant negative correlation between internet addiction and self-esteem, with addiction increasing as self-esteem decreased. However, there was a positive correlation between internet addiction and narcissism, with the number of self-portrait photographs on Facebook also showing a positive relationship. This suggests that internet addiction is a potentially serious public health problem, highlighting the need for further research on its impact on mental health [7]. The study addresses the growing global popularity of "Selfie Culture", investigating why selfies are increasingly posted on social media and who engages in this behavior. In this study, 60 college students in Delhi were interviewed, revealing five factors motivating offline selfie-taking (looks good, keeping memories, mood-driven, mirroring the self and social media posting) and three factors driving online posting (social approval, wanting to stand out and maintaining online presence). The findings highlight the need for further exploration of offline selfie practices and contribute to a better understanding of the selfie phenomenon [8]. A descriptive study investigates selfie-related behavior by assessing the SBS among 490 Italian participants, analyzing its links to dark triad personality traits and social media addiction. The SBS demonstrated solid psychometric properties and revealed a five-factor structure. The research found that selfitis behavior mediates the relationship between narcissism and psychopathy and social media addiction, while Machiavellianism showed no connection to either. The results clarify previous inconsistencies in the association between dark triad traits and social media addiction, highlighting selfitis behavior as a crucial mechanism and shedding light on personality traits linked to social media co-dependence [9]. Teaching strategies focusing on minimizing selfie addiction behaviour have been explored by one study conducted in Indore, India [10]. Therefore, this study hypothesized that Mental health pathway can provide awareness regarding selfie-addiction and help overcome the same.

## Methodology:

## Research design and sample:

This was a quasi-experimental pretest posttest only study conducted on students at a selected college in Coimbatore, Tamil Nadu and India. Sample population comprised of students from three departments of the college, viz., Computer applications, accounting and finance and professional accounting. Students in the first year of college between 19-20 years old and those who had adequate internet facility were included in the study. Students who were absent during data collection were excluded from the study. The conceptual framework of the study was adapted from general framework model [10], which includes an input, in this study the variables assessed like demography, frequency and nature of selfie and selfie addiction behavior scale, a throughput that is the selfie addiction strategies employed, finally giving an output comprising the post-test assessment.

## Ethical considerations:

The study was approved by Institutional Human Ethics Committee (IHEC) (Project No. 20/223), dated September 24, 2020 and informed consent was obtained from students.

## Data collection:

The study was conducted on a virtual meeting platform (Zoom Cloud and Google Classroom meeting). After explaining the study objectives and obtaining informed consent from the students, a pre-test assessment was done in the first week, where demographic, frequency of selfie addiction and Selfitis Behavior Scale were administered. The sample was divided in groups of 30-35 for intervention. Each day data was collected 1 hour in the morning and afternoon. Data collection was done for 12 hours per week, including 18 Zoom cloud meetings and 2 Google form assessments. The post-test assessment was conducted after 5 weeks of teaching intervention. Data was collected of between 22nd March 2021 to 30th April 2021.

## Results:

**Figure 1:**
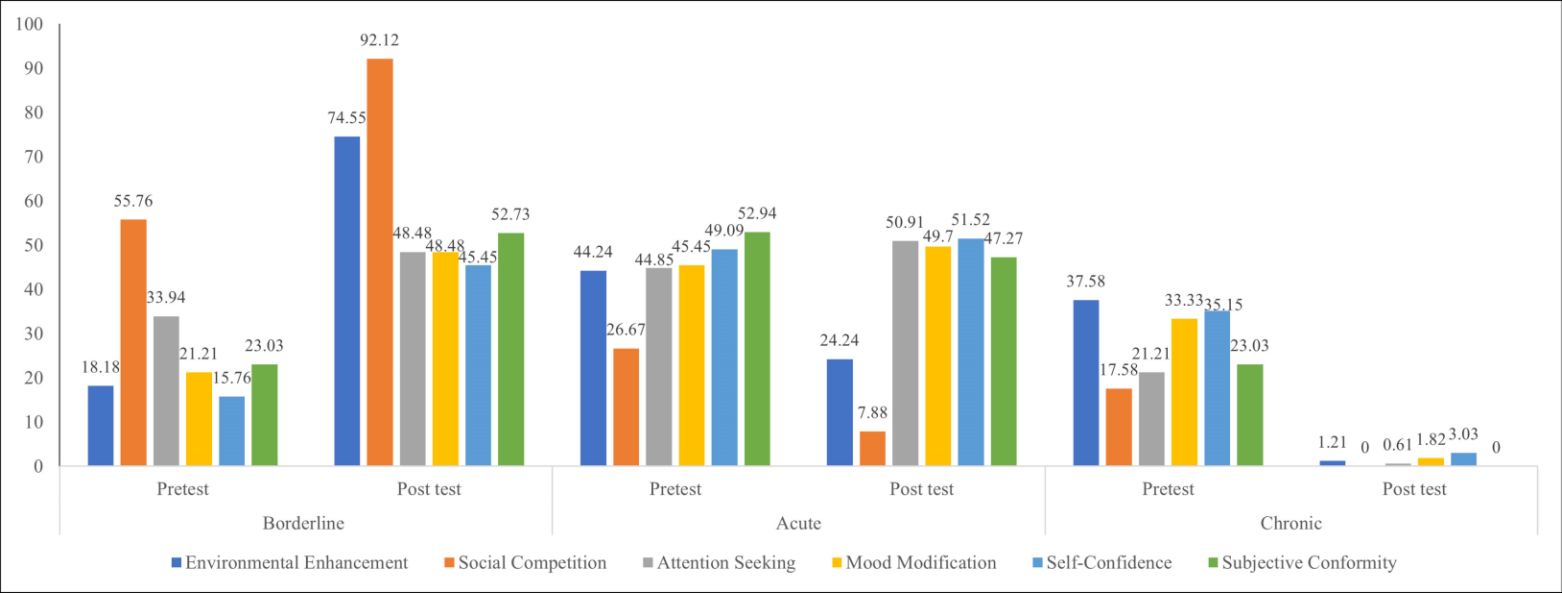
Percentage distribution of domain wise selfie addiction behavior among college students

Table 1 shows that Effectiveness of selfie addiction strategies: Mean Selfitis Behavior Scale scores when compared pre-test (57.20 18.44) and post-test (38.35 10.69), revealed a significant reduction in mean scores post-test (t=12.119, p=0.0001), highlighting the effectiveness of teaching and awareness sessions in reducing selfie-addiction behaviour. Similarly, domain-wise mean Selfitis Behavior Scale scores were compared pre-test and post-test. Reduction in mean scores were significant (p=0.0001) for each domain. The association of demographic variables with intensity of selfie addiction behaviour: On evaluating association of demographic variables with intensity of selfie addiction pre-test, a significant association was observed between gender and intensity (p=0.001), however post-test no significant association was observed (p=0.738). No association was found between other demographic variables and selfie addiction intensity, both pre and post-test. The association of intensity of selfie addiction behaviour with frequency and nature of selfies: Among frequency and nature of selfies variables pre-test, significant association with intensity of selfie addiction behaviour was observed with time taken for a selfie (p=0.029), number of selfies clicked per day (p=0.0001), whether group selfies were taken (p=0.005), where the selfies are posted (p=0.007) and filters used on selfies (p=0.013) and post-test no significant association was observed with any of these variables. The comparison of selfie addiction behaviour domains used the Bonferroni pairwise comparison of selfie addiction behaviour domains revealed highly significant reduction in levels of environmental enhancement and social competition domains (p<0.0001). Figure 1 shows that Percentage distribution of domain wise pre and post-test level of selfie addiction behavior among college students. Environmental Enhancement showed a notable shift with chronic cases dropping from 37.58% to 1.21% while borderline cases rose from 18.18% to 74.55%. The most striking change occurred in Social Competition, where chronic cases were eliminated (17.58% to 0%) and borderline cases increased substantially from 55.76% to 92.12%. Similar positive trends were observed in the remaining domains: Attention Seeking saw chronic cases decrease to 0.61% from 21.21%, Mood Modification's chronic cases fell from 33.33% to 1.82%, Self-Confidence domain showed reduction in chronic cases from 35.15% to 3.03% and Subjective Conformity achieved complete elimination of chronic cases. All domains demonstrated a clear pattern of movement from severe to milder forms of addiction, with consistent increases in borderline cases suggesting successful behavioral modification.

## Discussion:

The present study revealed pre-test post-test methodology, this study confirms the effectiveness of the selfie addiction strategies employed to reduce or prevent selfie addiction behaviour, that is evident by significant reduction in mean Selfitis Behavior Scale intensity scores (p=0.0001) and mean domain-wise scores post-test compared to pre-test (p=0.0001). Comparing with the only other study conducted in Indore [10], a structured teaching program significantly increased the knowledge score post-test. However, the authors did not employ any selfie-addiction assessment scales. This points out to a need to disseminate teaching techniques in order to create awareness about addiction behaviour associated with selfie taking. There was significant association of gender with intensity of selfie addiction in this study (p=0.0001), where more female students exhibited selfie addiction behaviour pre-test. The reason for this could be significant body image dissatisfaction, concerns over appearance and worsening moods among females [11]. This finding was, however, reduced post-test. The study findings also reveal significant association of frequency and nature of selfies, especially time taken for a selfie (p=0.029), number of selfies clicked per day (p=0.0001), whether group selfies were taken (p=0.005), where the selfies are posted (p=0.007) and filters used on selfies (p=0.013), with intensity of selfie addiction behaviour. A similar kind of descriptive study with 60 engineering students, revealing 15% mild, 68% moderate and 17% severe selfitis behavior. They found significant associations between emotional state and family type (chi-square=37.65, t=15.45, p<0.05), emphasizing the role of demographic factors in addiction patterns [12]. Similarities along the lines of frequency of selfies was observed in studies where majority of them took and posted one (51.7%) and 1-4 selfies (90.9%) at least every day [13] and 82.4% participants of this study took at least 0-2 selfies in a day. While a mere 2.4% of 165 participants in this study took selfies to post on social media, study conducted in Eastern India reported 40.3% uploaded selfies on social media every day [5]. Another supportive cross-sectional study with 273 Allied Health Sciences students found significant relationships between selfie addiction and various demographic factors (p<0.05). Their research revealed higher addiction rates among urban students (44.3% moderate level) and first-year students (38.0% moderate level) [14]. A pairwise comparison of domains of Selfitis Behavior Scale saw a highly significant reduction in levels of environmental enhancement and social competition. Taking selfies at an enjoyable environment is known to make lasting memories [9], but during a short period of time between 2014 - '16, 75 deaths due to selfie have been identified worldwide, with India witnessing the greatest number of deaths (41), in 52 separate incidents including falling from heights, drowning and rail accidents. Social competition aids in creativity to post more creative content than other online friends and acquaintances, getting them social recognition and acceptance [15]. These findings emphasize the importance of a holistic approach to improve educational practices and support student needs through technology [16]. A similar kind of correlational study with 100 college students, finding no significant gender differences in addiction scores (t=1.395, p>0.05), contrasting with our findings. They reported a strong correlation between selfie addiction and academic performance (r=0.260, p<0.001), suggesting negative academic impacts [17]. The consequences of selfie addiction are perceived as illusory and threatening to self-esteem, even though self-presentation increases confidence among people [18]. Although "selfitis" has not been recognized as an addiction disorder [19], the current findings along with other studies highlight the prevalence of excessive selfie taking, resulting in addiction behaviors. Further, studies should also focus on association of frequency and nature of selfies with intensity of addiction behavior, which can help in charting out selfie "hygiene" measures to prevent any addictive behaviors.

## Conclusion:

Data shows that female students exhibited greater selfie addiction influenced by selfie frequency and type. Teaching strategies effectively reduced addiction linking it to environmental and social factors.

## Figures and Tables

**Table 1:** Effectiveness of selfie addiction domain-wise selfie addiction behaviour scale scores pre-test and post-test among college students

**Selfie Addiction Behaviour Domains**	**Test**	**Mean ± SD**	**Paired t test and p value**
Environmental Enhancement	Pre-test	13.73 ± 3.57	t = 15.485,
	Post-test	8.76 ± 2.94	p = 0.0001*
Social Competition	Pre-test	10.38 ± 4.16	t = 8.145,
	Post-test	7.48 ± 2.23	p = 0.0001*
Attention Seeking	Pre-test	7.47 ± 3.38	t = 6.830,
	Post-test	5.45 ± 1.89	p = 0.0001*
Mood Modification	Pre-test	8.66 ± 3.62	t = 10.256,
	Post-test	5.59 ± 2.03	p = 0.0001*
Self-Confidence	Pre-test	9.08 ± 3.29	t = 11.333,
	Post-test	5.75 ± 2.25	p = 0.0001*
Subjective Conformity	Pre-test	7.88 ± 3.14	t = 9.166,
	Post-test	5.33 ± 1.92	p = 0.0001*
***indicates p<0.001 Significant
